# Supplementary figures and images for: Sustained ErbB Activation Causes Demyelination and Hypomyelination by Driving Necroptosis of Mature Oligodendrocytes and Apoptosis of Oligodendrocyte Precursor Cells
Source: J Neurosci. 2021 Dec 1;41(48):9872–90. doi: 10.1523/JNEUROSCI.2922-20.2021 (PMC8638686; doi:10.1523/JNEUROSCI.2922-20.2021)

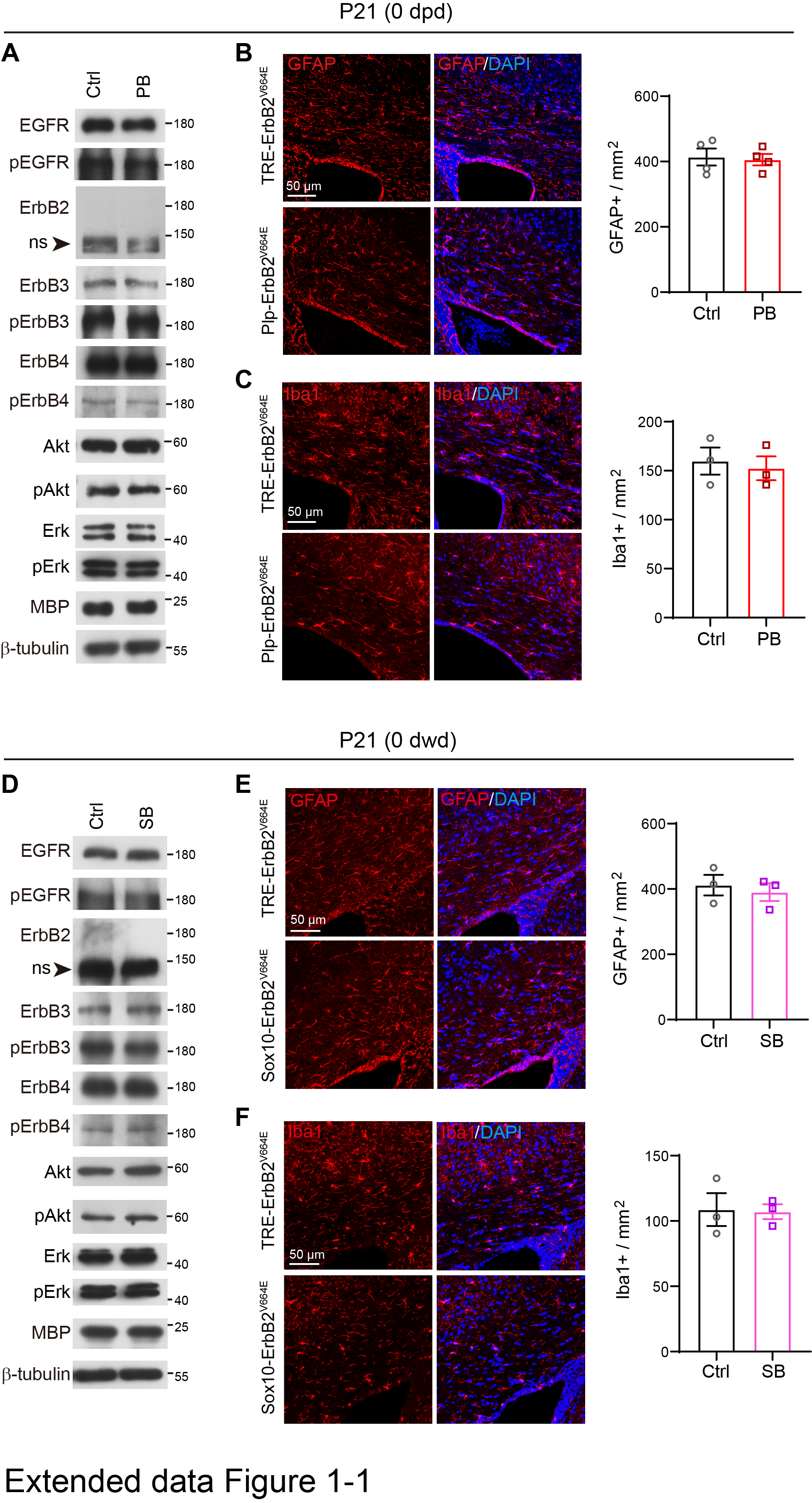

Supplement: Extended Data Figure 1-1 — No leak in transgene expression in mice at P21 before drug induction. A, D, Western blotting of indicated proteins in white matter isolated from Plp-ErbB2V664E (PB) mice and littermate control mice (Ctrl) at P21 before Dox withdrawal (A), or from Sox10-ErbB2V664E (SB) mice and littermate control mice (Ctrl) at P21 before Dox treatment (D). ns, nonspecific bands. B, C, E, F, Astrocytes (GFAP+) and microglia (Iba1+) examined in the corpus callosum of indicated mice by immunostaining. Cell densities in the corpus callosum were quantified, and data were presented as mean ± SEM. Statistical analyses by unpaired t test revealed no differences. Download Figure 1-1, TIF file. [file ns-JN-RM-2922-20-s01.tif]

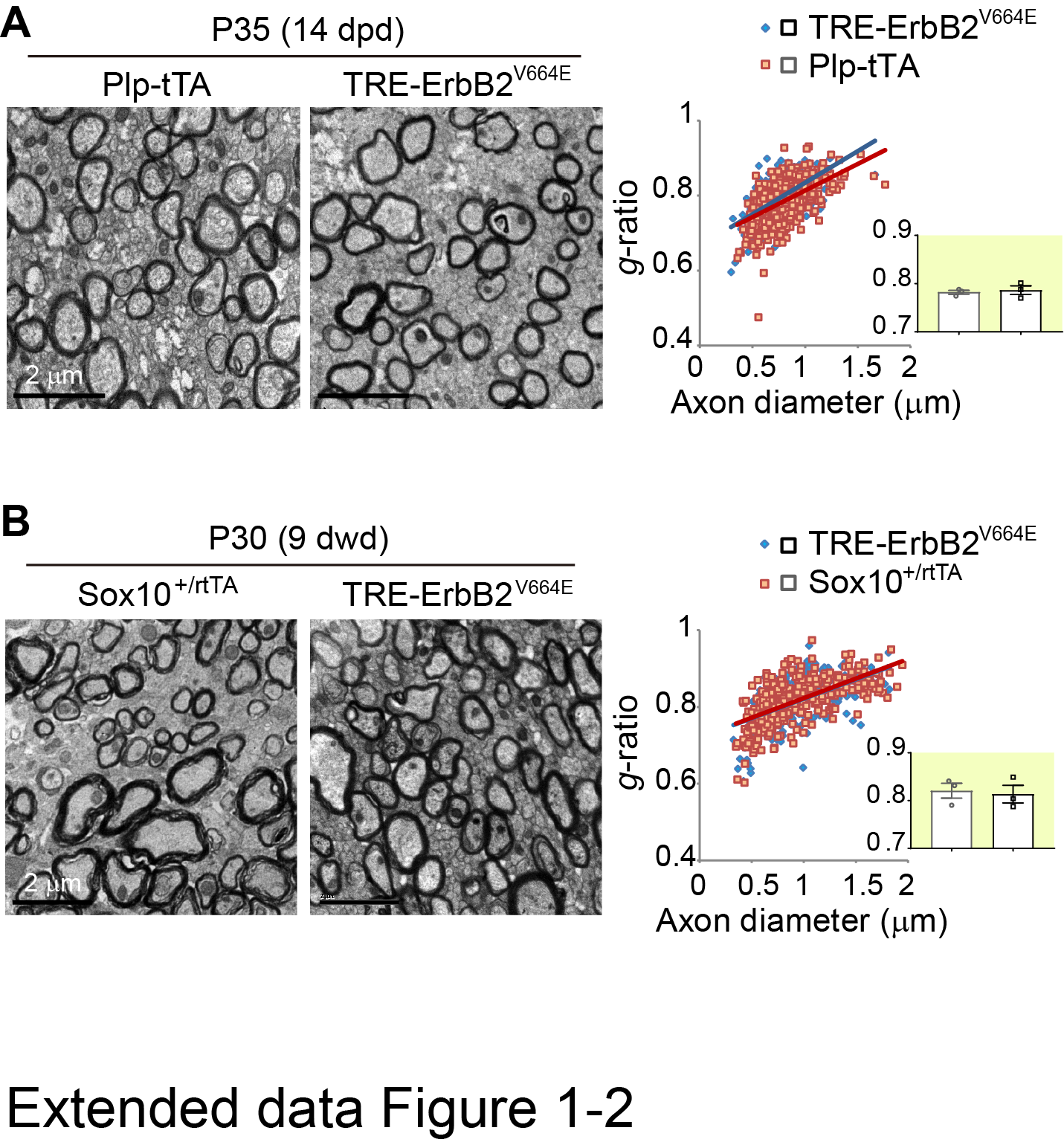

Supplement: Extended Data Figure 1-2 — Unaltered myelin in the brains of Plp-tTA or Sox10+/rtTA mice after Dox treatments. A, B, EM images of the corpus callosum of Plp-tTA and littermate TRE-ErbB2V664E mice at P35 with 14 dpd (A), or that of Sox10+/rtTA and littermate TRE-ErbB2V664E mice at P30 with 9 dwd (B). g-ratio was calculated for myelinated axons. Averaged g-ratio (inset) were presented as mean ± SEM and analyzed by unpaired t test. For A, t(4) = 0.4472, p = 0.678; for B, t(4) = 0.3042, p = 0.776. Download Figure 1-2, TIF file. [file ns-JN-RM-2922-20-s02.tif]

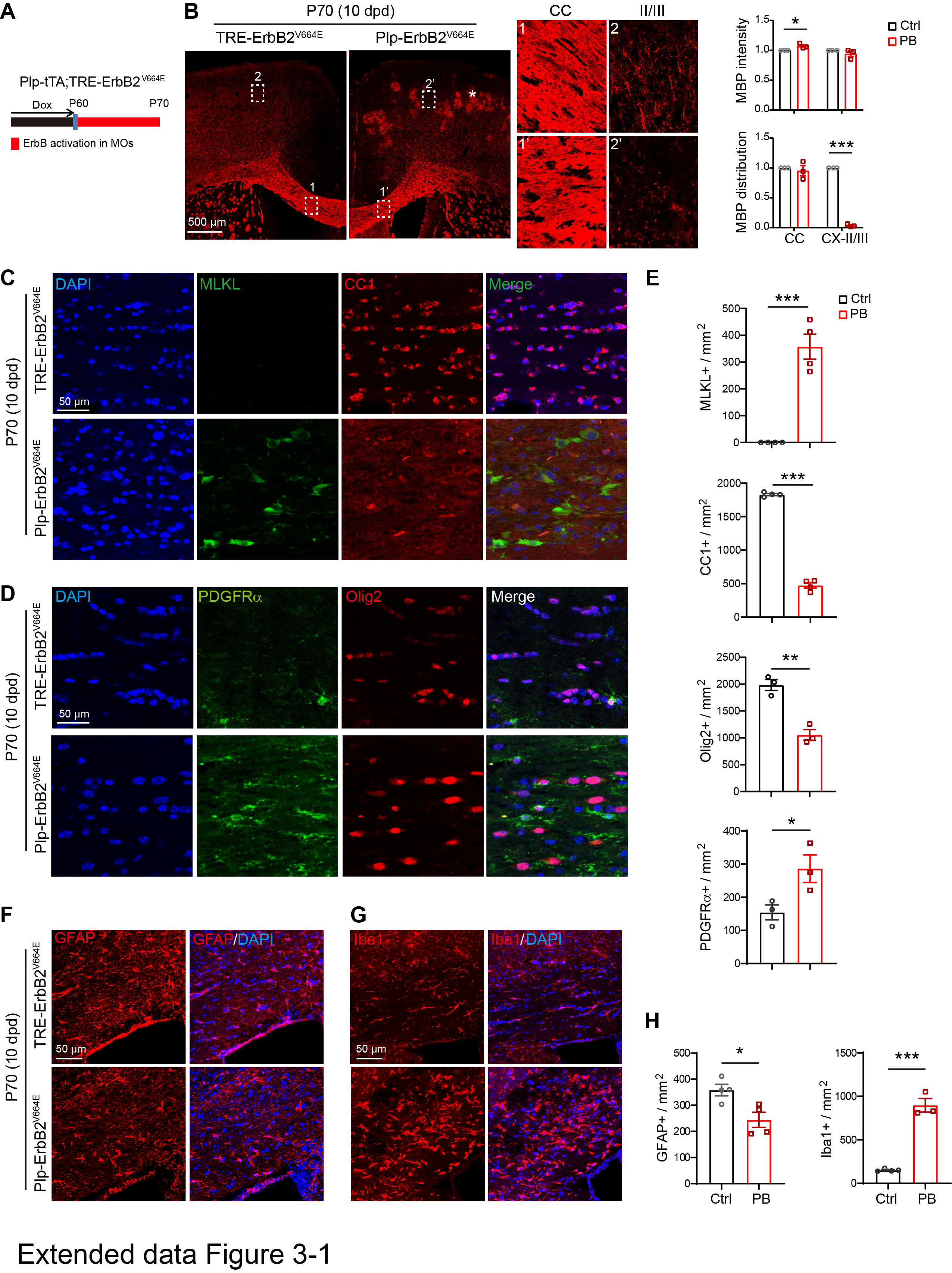

Supplement: Extended Data Figure 3-1 — ErbB overactivation induced pathologic changes in white matter of adult Plp-ErbB2V664E mice. A, Dox treatment setting for indicated mice and littermate controls. B, MBP immunostaining of Plp-ErbB2V664E mice and littermate controls at P70 with 10 dpd. Magnified regions exhibited that myelin structures were comparable in the corpus callosum (CC; 1 and 1') whereas became fragmented and beaded in Layer II/III of the cortex (II/III, 2 and 2') in Plp-ErbB2V664E mice. Note there were more nonspecifically stained hemorrhagic spots (white asterisk) in the cortex of these mice than that of Plp-ErbB2V664E mice with similar treatment at P30 with 9 dpd. Quantitative data were presented as mean ± SEM and analyzed by unpaired t test. For MBP intensity of CC, t(4) = 3.033, p = 0.0386; CX-II/III, t(4) = 1.204, p = 0.295. For MBP distribution of CC, t(4) = 0.5432, p = 0.616; CX-II/III, t(4) = 67.58, p < 0.0001. C, D, Representative immunostaining results of MLKL, CC1, PDGFRα, Olig2 in the CC of Plp-ErbB2V664E and littermate control mice at P70 with 10 dpd. E, Quantitative data of oligodendrocyte densities in the CC were presented as mean ± SEM and analyzed by unpaired t test. For MLKL+, t(6) = 7.716, p = 0.0002; for CC1+, t(6) = 31.34, p < 0.0001; for Olig2+, t(4) = 6.372, p = 0.0031; for PDGFRα+, t(4) = 2.8, p = 0.0488. F, G, Astrocytes (GFAP+) and microglia (Iba1+) examined in the subcortical white matter of indicated mice by immunostaining. H, Astrocyte and microglia densities in the CC were quantified, and data were presented as mean ± SEM and analyzed by unpaired t test. For GFAP+, t(6) = 3.148, p = 0.0199; for Iba1+, t(5) = 11.36, p < 0.0001. Note the GFAP+ cell densities were not increased, despite that of Iba1+ cells increased dramatically, indicating a different inflammatory profile in adult mice. Download Figure 3-1, TIF file. [file ns-JN-RM-2922-20-s03.tif]

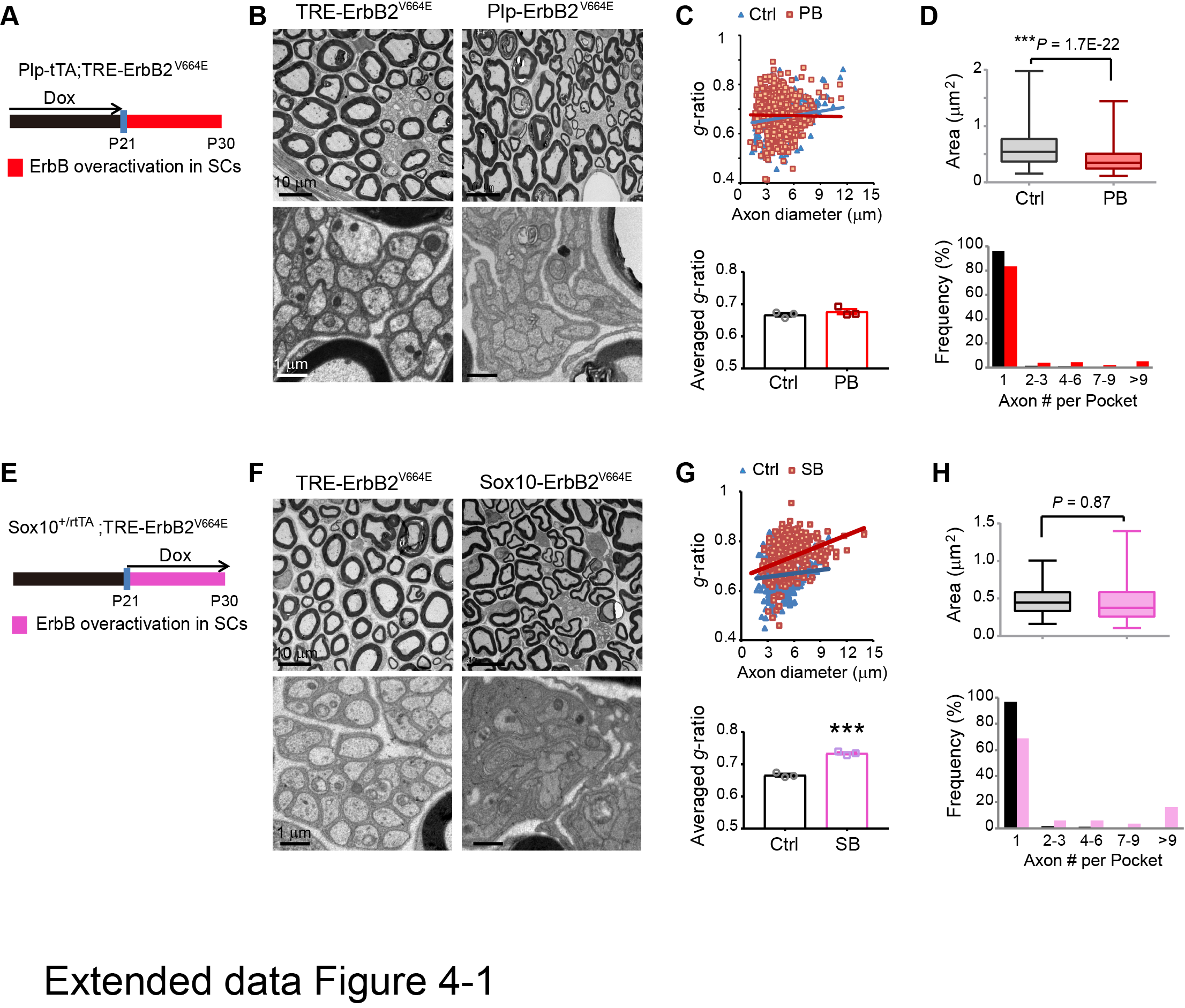

Supplement: Extended Data Figure 4-1 — ErbB overactivation induced hypomyelination of peripheral nerves in Sox10-ErbB2V664E mice but not in Plp-ErbB2V664E mice. A, E, Dox treatment setting for indicated mice and littermate controls. SCs, Schwann cells. B, F, Representative EM images of sciatic nerves for Plp-ErbB2V664E with littermate controls (B), or Sox10-ErbB2V664E with littermate controls (F). The top layers of EM images showed myelinated axons in the sciatic nerves, while the bottom layers of EM images showed unmyelinated axons. C, G, Quantitative data were shown for g-ratio analysis of myelinated axons detected by EM. Averaged g-ratio for each mouse were plotted at the bottom, presented as mean ± SEM, and analyzed by unpaired t test. For C, t(4) = 1.026, p = 0.363; for G, t(4) = 12.63, p = 0.0002. D, H, Both Plp-ErbB2V664E and Sox10-ErbB2V664E mice exhibited slight deficiency in unmyelinated axons of the sciatic nerves. Top, Axonal sizes of unmyelinated axons were measured by their areas in cross sections, and data were plotted as boxes showing quartile and median with whiskers to show 2.5–97.5% of data range. Outlier symbols were omitted. Data were analyzed by unpaired t test. Bottom, For the ensheathment analysis of unmyelinated axons, axon numbers in each pocket were counted and quantified by their frequency. For normal mature peripheral nerves, the majority of nonmyelinating SC pockets only ensheathe one axon. Download Figure 4-1, TIF file. [file ns-JN-RM-2922-20-s04.tif]

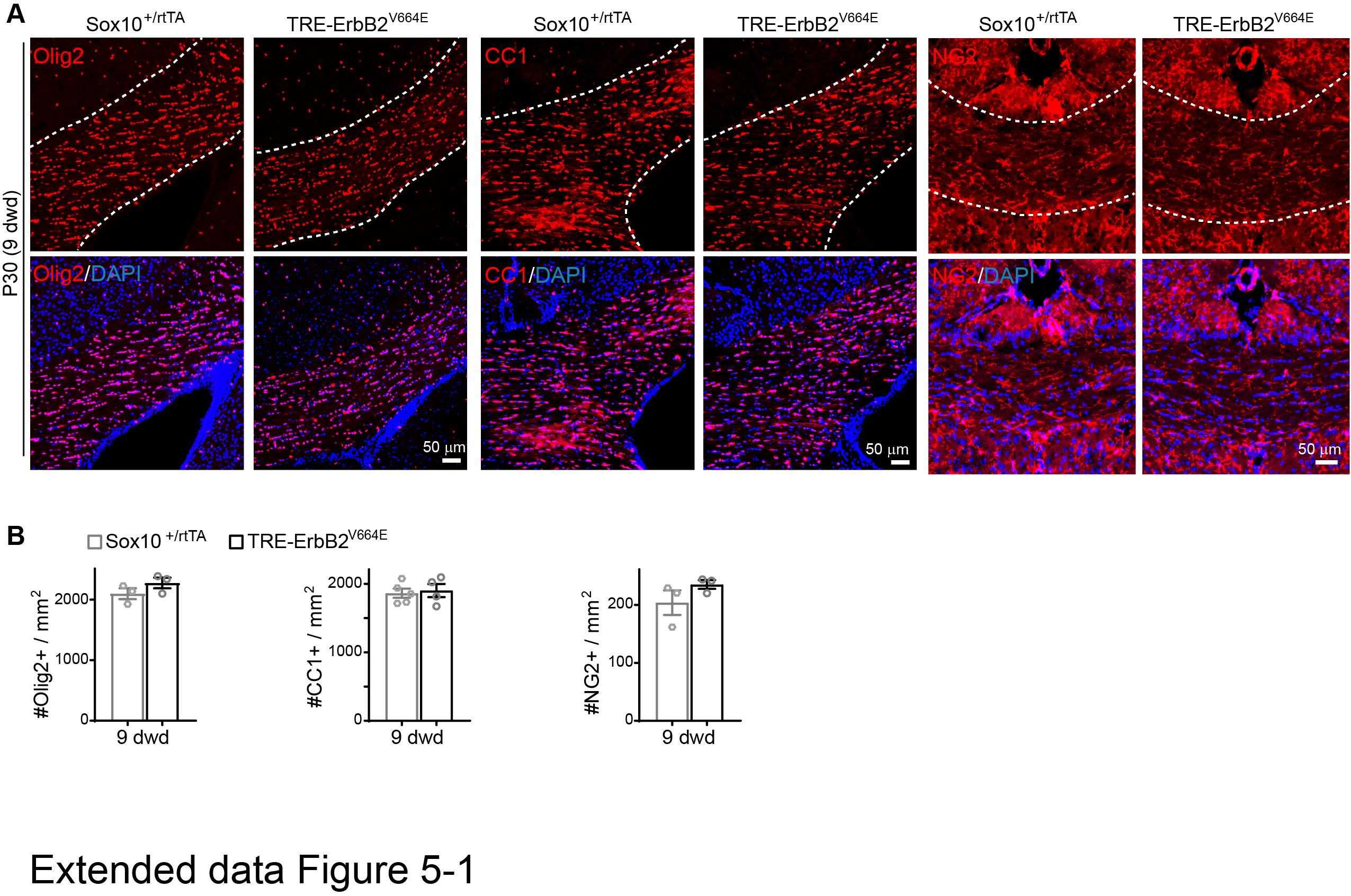

Supplement: Extended Data Figure 5-1 — Oligodendrocyte cell densities were similar in the corpus callosum of Sox10+/rtTA and littermate TRE-ErbB2V664E mice. A, Olig2+, CC1+, and NG2+ cells in the corpus callosum of indicated mice at P30 with 9 dwd were examined by immunostaining. B, Data were from immunostaining of three mice for each group, presented as mean ± SEM, and analyzed by unpaired t test. For Olig2+, t(4) = 1.418, p = 0.229; for CC1+, t(7) = 0.3431, p = 0.742; for NG2+, t(4) = 1.394, p = 0.236. Download Figure 5-1, TIF file. [file ns-JN-RM-2922-20-s05.tif]

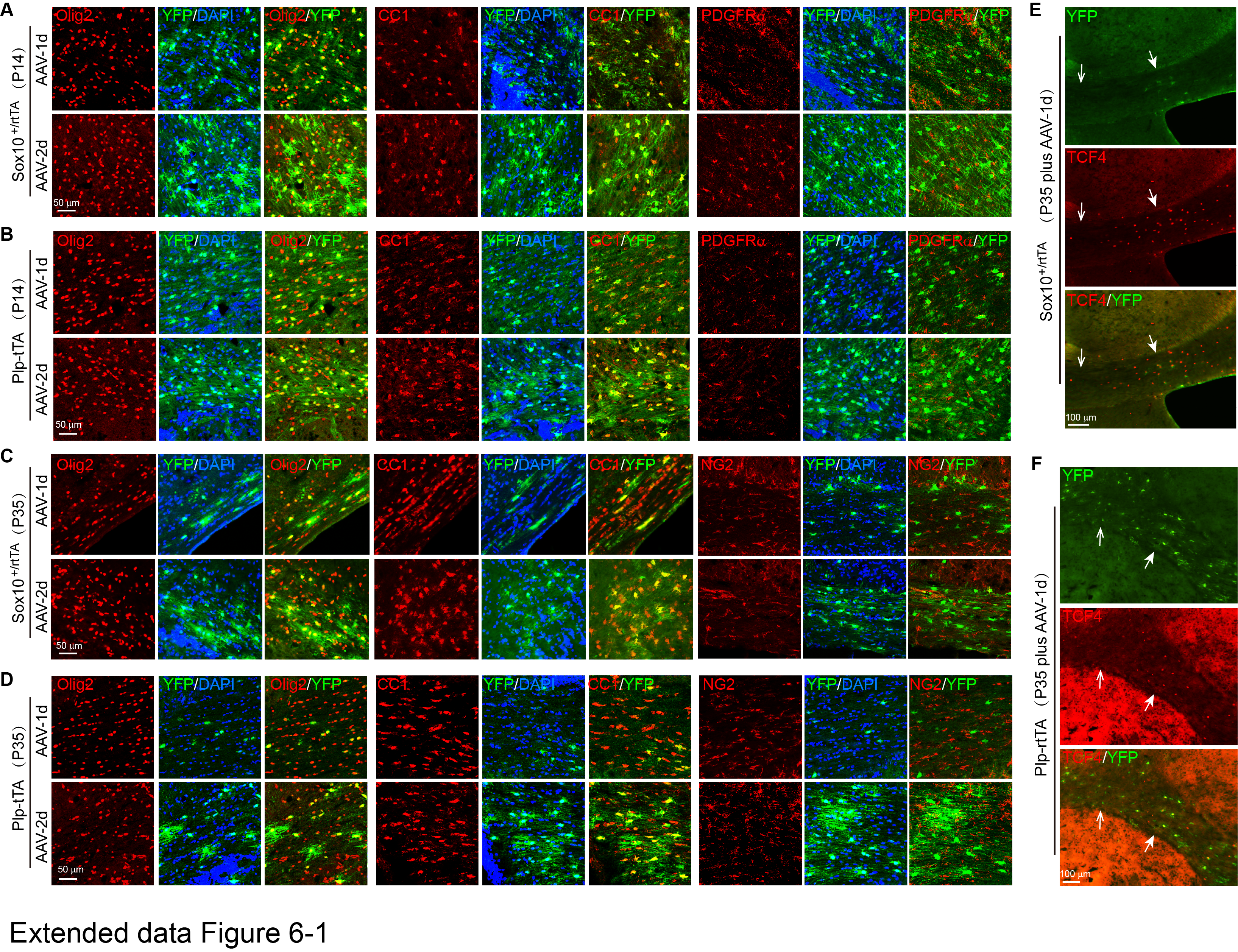

Supplement: Extended Data Figure 6-1 — Pulse-labeled reporter-containing oligodendrocytes in Plp-tTA and Sox10+/rtTA at P14 or P35. A–D, AAV-TRE-YFP was stereotactically injected into the corpus callosum of Sox10+/rtTA or Plp-tTA mice at P14 or P35. One (AAV-1d) or two (AAV-2d) days after virus injection, brain sections were co-immunostained by antibodies to YFP and Olig2, or by CC1 antibody and antibody to YFP, or by antibodies to YFP and NG2 (or PDGFRα). Shown are representative images for indicated mice at P14 or P35. Sox10+/rtTA mice were fed with Dox for 3 d before stereotaxic injection of the virus, while Plp-tTA mice had no Dox treatment. E, F, Distributions of viral pulse-labeled cells, as shown by co-immunostaining of YFP and TCF4, in the corpus callosum of Sox10+/rtTA (E) or Plp-tTA (F) mice at P35. Note that reporter-containing (YFP+) cells in Sox10+/rtTA mice stringently distributed within TCF4+ cell clustered region, whereas those in Plp-tTA mice distributed broadly in the corpus callosum. Solid arrows, regions with clustered TCF4+ cells; open arrows, regions with fewer TCF4+ cells. Download Figure 6-1, TIF file. [file ns-JN-RM-2922-20-s06.tif]
